# Authors Response: Tuberculosis and pulmonary candidiasis co-infection present in a previously healthy patient

**Published:** 2016-09-30

**Authors:** Dilia Mildret Fontalvo, Gustavo Jiménez Borré, Doris Gómez Camargo, Neylor Chalavé Jiménez, Javier Bellido Rodríguez, Bernarda Cuadrado Cano, Shirley Navarro Gómez

**Affiliations:** 1 Departamento de Postgrado, Doctorado en Medicina Tropical, Universidad de Cartagena, Cartagena, , Colombia; 1 Grupo de investigación UNIMOL. Universidad de Cartagena, Cartagena, Colombia; 1 Unidad de Cuidados Intensivo Adultos, Departamento de Medicina Interna, Clinica Nuestra. Cartagena, Colombia

## Dear Editor:

Article ref: https://www.ncbi.nlm.nih.gov/pubmed/27546933

In response to the note 1 about the case described 2, we fully agree that immunodeficiency is not only the relationship with HIV infection, and that there are pathologies and different immunological and genetic conditions associated with it 3-5; the main ones were discarded in the patient. 

In the patient of the presented clinical case, there is no family history of primary immunodeficiencies. And in her personal history, there were not found any data related to recurrent infectious processes, either in childhood or present, which does not lead to suspicion of diseases with primary immunodeficiencies, in which recurrent infections would be expected as in the case of recurrent pneumonia, lung, spleen and liver abscesses, cervical, axillary and inguinal lymphadenitis, or bone and skin infections, as in the case of chronic granulomatous disease 6. 

For other primary immunodeficiencies provided by the reader, such as the case of X-linked agammaglobulinemia, this is a congenital disease that affects males and involves B lymphocytes and plasma cells, which are not the primary immune line in tuberculosis 7 ^6^, nor does it correspond to our case. 

On the other hand, inherited immune system defects, such as Mendelian susceptibility to mycobacterial diseases syndromes (MEMS), in which there are defects in the axis IL-12/IN-γ, can be a major cause of fungal and mycobacterial associations, as in the patient of the clinical case; although it would be expected that these patients present, since their birth, a history of oral, skin and enteral fungal infections, with a very important fact, as it is the presence of axillary lymphadenitis and disseminated mycobacterial infection with the implementation of the BCG vaccine, and pigmented purpuric dermatosis 8, data that were not found in our patient. 

Within the recorded history, we found out that she was not receiving any medication related to immunosuppression. Studies to rule out metabolic, kidney and liver diseases were performed, including arterial blood gases, serum electrolytes, protein electrophoresis, coagulation tests, quantification of serum immunoglobulins, studies of renal function (urinalysis and urinary sediment, creatinine, BUN) and hepatic function (bilirubin, alanine transaminase, aspartate transaminase, alkaline phosphatase, serum albumin, prothrombin time), all of which were normal. Elisa test for HIV was negative. For the purpose of seeking collagen pathologies, antinuclear and anti-double-stranded DNA antibodies were performed, with negative results. 

With respect to macrocytic anemia in the initial blood count at the admission of the patient, there were no data of personal or family history of anemia, and this condition was corrected during ambulatory evolution, suggesting a case of possible infectious condition. 

Checks performed in the outpatient patient reveal that she is evolving satisfactorily. She is on medical supervision for internal medicine and infectious diseases under her health insurer, where he underwent blood count, serological determinations of IgA, IgG, IgE, CD4 and CD8, all of which were normal. 

In this case, both clinical and para-clinical follow-up was definitive to determine associations with underlying conditions as predisposing factors for the coexistence of tuberculosis and pulmonary candidiasis. 

However, clinical cases are an invitation to seek scientific explanations, to think on these clinical entities; in addition, they can give some guidelines to guide us in other similar situations, and to generate us concerns about the pathogenesis of primary immunodeficiencies, and the possible monogenic or other genetic defects to explain these susceptibilities. 

Nevertheless, we have found very good and important the questioning and exhortation that the author does, and that we do and extend to all clinical colleagues: We must carefully use the term immunocompetence when we study a patient, and to perform an optimal evaluation to those who present with opportunistic infections. 

The authors express their gratitude for this important contribution. 
